# Theoretical Analysis of Competing Conformational Transitions in Superhelical DNA

**DOI:** 10.1371/journal.pcbi.1002484

**Published:** 2012-04-26

**Authors:** Dina Zhabinskaya, Craig J. Benham

**Affiliations:** UC Davis Genome Center, University of California, Davis, California, United States of America; Ottawa University, Canada

## Abstract

We develop a statistical mechanical model to analyze the competitive behavior of transitions to multiple alternate conformations in a negatively supercoiled DNA molecule of kilobase length and specified base sequence. Since DNA superhelicity topologically couples together the transition behaviors of all base pairs, a unified model is required to analyze all the transitions to which the DNA sequence is susceptible. Here we present a first model of this type. Our numerical approach generalizes the strategy of previously developed algorithms, which studied superhelical transitions to a single alternate conformation. We apply our multi-state model to study the competition between strand separation and B-Z transitions in superhelical DNA. We show this competition to be highly sensitive to temperature and to the imposed level of supercoiling. Comparison of our results with experimental data shows that, when the energetics appropriate to the experimental conditions are used, the competition between these two transitions is accurately captured by our algorithm. We analyze the superhelical competition between B-Z transitions and denaturation around the *c-myc* oncogene, where both transitions are known to occur when this gene is transcribing. We apply our model to explore the correlation between stress-induced transitions and transcriptional activity in various organisms. In higher eukaryotes we find a strong enhancement of Z-forming regions immediately 5′ to their transcription start sites (TSS), and a depletion of strand separating sites in a broad region around the TSS. The opposite patterns occur around transcript end locations. We also show that susceptibility to each type of transition is different in eukaryotes and prokaryotes. By analyzing a set of untranscribed pseudogenes we show that the Z-susceptibility just downstream of the TSS is not preserved, suggesting it may be under selection pressure.

## Author Summary

The stresses imposed on DNA within organisms can drive the molecule from its standard B-form double-helical structure into other conformations at susceptible sites within the sequence. We present a theoretical method to calculate this transition behavior due to stresses induced by supercoiling. We also develop a numerical algorithm that calculates the transformation probability of each base pair in a user-specified DNA sequence under stress. We apply this method to analyze the competition between transitions to strand separated and left-handed Z-form structures. We find that these two conformations are both competitive under physiological environmental conditions, and that this competition is especially sensitive to temperature. By comparing its results to experimental data we also show that the algorithm properly describes the competition between melting and Z-DNA formation. Analysis of large gene sets from various organisms shows a correlation between sites of stress-induced transitions and locations that are involved in regulating gene expression.

## Introduction

DNA structure has been known to be polymorphic since the earliest days of its investigation. Rosalind Franklin in her initial fiber diffraction studies found two distinct DNA structures, which she called the A-form and the B-form [1]. Transitions between these forms could be induced by changes of hydration state, with B-DNA being the hydrated form and hence presumably the biologically relevant structure. Yet the first determination of DNA structure at atomic resolution found that the 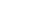 sequence crystallizes into a left-handed helix, called Z-DNA [2].

Although the standard right-handed B-form helix is known to be the prevalent structure *in vivo*, DNA can assume many other conformations. Some, such as the A-form and the strand separated state, can occur in any DNA sequence, although the latter prefers A+T-rich sequences. Other conformations have either specific sequence requirements or strong preferences for certain sequence types. These include the Z-form, which prefers alternating purine-pyrimidine sequences, the cruciform, which requires a high degree of inverted repeat symmetry, the triple stranded H-form, which needs long, mirror symmetric homopurine or homopyrimidine runs, and the four stranded G-quadriplex structure, which requires four runs of G's in close proximity.

Transitions from B-form to alternate DNA structures can be induced in susceptible sequences in a variety of ways, including changes of temperature, ionic conditions, hydration, or superhelical state. The first three of these conditions are approximately constant *in vivo*; only the level of imposed DNA superhelicity is subject to physiological changes that can affect the propensity of the molecule to transform to alternate conformations. Substantial levels of negative superhelicity are imposed on DNA *in vivo* by gyrase enzymes in prokaryotes, and by transcriptional activity in all organisms [3]. Although the superhelicity imposed by transcription in eukaryotes is transient, it is known to travel over kilobase distances and to persist long enough to drive DNA structural transitions [4].

Negative superhelicity imposes undertwisting torsional stresses on the DNA, which can induce transitions to alternate conformations that are less twisted in the right-handed sense than is B-DNA. Transitions to such states decrease the local helical twist, and thereby relieve some of the imposed superhelical stress. A transition will become favored at equilibrium when the amount of stress energy it relieves exceeds its energy cost. *In vitro* experiments have demonstrated superhelical transitions from the B-form to each of several types of alternate structures, including Z-DNA [5], [6], H-DNA [7], locally strand separated DNA [8], [9], and cruciforms [10]–[12]. A structural transition also has been observed to occur in a superhelical plasmid that contains a quadriplex-susceptible region, although it was not verified that the alternate structure involved is the quadriplex [13]. It has been suggested that this region instead prefers to form H-DNA, to which it also is susceptible [14].

Since genomic DNA often has numerous sites whose sequences are susceptible to forming these alternate structures, in principle there are many different combinations of transitions that can occur in response to imposed negative superhelicity. Moreover, the transition behavior of each susceptible site is coupled to the behaviors of all other sites that experience the same superhelical stress. This coupling occurs because each transition relieves some of the imposed stress, which alters the probability of transition at all other sites throughout the region involved. In this way imposed superhelicity induces a global competition among all the sites that are susceptible to any type of transition. Non-linear and highly complex correlations occur among the transition behaviors of susceptible regions throughout the domain. To analyze this competition in its full complexity, it is necessary to develop methods that can treat the simultaneous occurrence of multiple competing transitions of different types. This paper presents the first computational method to analyze competing superhelical transitions in this way.

Several theoretical models have been developed previously to analyze superhelical transitions in DNA. The earliest models were mechanical in nature, treating the transition as an “on-off” mechanism at a single susceptible site within a sequence that was otherwise unable to transform [15]–[18]. Subsequently, more detailed models were developed that used statistical mechanics to analyze transitions at a single susceptible site in a transition-resistant background [19]–[21]. This strategy is the basis for the *Z-Hunt* algorithm, which searches for individual Z-susceptible regions within a sequence by assessing the ability of each to undergo transition when placed alone in an otherwise non-transforming plasmid [22]. Competitions between two susceptible sites within a non-transforming background were also treated in this way. In some cases these involved two sites susceptible to the same type of transition [18], [23], [24]. In others idealized competitions between different types of transitions were examined, such as cruciform extrusion vs B-Z transitions [18], [25] and denaturation vs B-Z transitions [16], [26]. Although these models illuminated basic properties of superhelical transitions, they did not include the full competition among multiple sites that can occur in genomic sequences.

It was soon recognized that a complete statistical mechanical treatment was required to accurately simulate the competitive behavior of conformational transitions in superhelical DNA sequences of kilobase lengths, which commonly contain numerous sites whose sequences render them imperfectly susceptible to transitions of several types. Several algorithms have been developed to analyze superhelical transitions to a single type of alternate structure in DNA sequences where every base pair is regarded as being able to assume that structure [27]–[31]. A conformational state is determined by specifying which base pairs are in the B-form state and which are in the alternate structure. These states are weighted according to the Boltzmann distribution, from which equilibrium properties of the system are determined under given environmental conditions and levels of supercoiling. This approach has been applied individually to each of several types of transitions, including strand separation and B-Z transitions, and a modified version has been used to treat cruciform extrusion [17], [30]–[32]. Some of the techniques that have been developed are formally exact but computationally very slow [30], while others are approximate. Among the approximate methods, the SIDD (stress-induced duplex destabilization) and the SIBZ (stress-induced B-Z transition) algorithms for treating strand separation and B-Z transitions, respectively, are based on a similar algorithmic strategy, which has proven to be both highly accurate and computationally efficient [31], [32].

In order to develop quantitatively accurate statistical mechanical methods, it is necessary to have detailed knowledge of the alternate conformations being analyzed. One must know the geometry and flexibility of each alternate conformation, the energies of junctions between that structure and others (most importantly the B-form), and the sequence-specific energetics of the transition from B-DNA to each conformation. Since strand separation and B-Z transitions have been implicated in biological functions, they have been widely studied. So the information regarding the energetics of these two transitions is available that enables their quantitative analysis. For this reason in this paper we focus on applying our multi-state approach to the competition between denaturation and B-Z transitions in superhelical molecules.

Local separation of the two DNA strands at the correct times and locations is necessary for the initiation of transcription and replication, two key functions of DNA. Superhelical strand separation was the first DNA transition to be rigorously modeled in a way that enabled the analysis of sequences having arbitrary lengths [28], [29], [32], [33]. The SIDD algorithm that was developed for this purpose has been applied to analyze a wide variety of DNA sequences, including complete genomes. Its results agree closely with experimental observations of the level of supercoiling required to drive stand separation and the locations of the melted regions within a sequence in all cases where experiments have been performed [29], [32], [34]–[36]. Since it costs less energy to melt an AT base pair than a GC base pair, local strand separation tends to occur in the A+T-rich regions of a sequence. Stress-induced duplex destabilization has been implicated in a variety of important biological processes, including the initiation of transcription from specific promoters, the functioning of replication origins in yeast and viruses, and scaffold attachment in eukaryotes [34]–[42].

Shortly after the discovery of Z-DNA it was theoretically predicted and experimentally verified that transitions to this structure could be driven by physiologically attainable levels of negative superhelicity [2], [16], [19], [20]. Z-DNA has been experimentally detected at inserted Z-susceptible regions in torsionally stressed bacterial DNA, both *in vitro* and *in vivo* [21], [43]–[47]. There is strong indirect evidence suggesting that Z-DNA also may occur in eukaryotic genomes *in vivo* [48]–[51]. At present, specific biological activities of Z-DNA have not been fully elucidated, although there is substantial indirect evidence that it may serve regulatory functions in several processes [48]. The repeat unit of Z-DNA is a dinucleotide, with one base pair in the *anti* and the other in the *syn* conformation. Although Z-DNA is known to prefer alternating purine-pyrimidine sequences, specifically 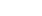 or 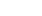 runs, it can occur in other base sequences at a higher energy cost [22]–[24], [43], [45], [52]–[54]. The junctions energies and the free energies of the B-Z transition have been determined for all ten dinucleotides, including their dependence on their *anti/syn* character [21], [22], [25], [52]–[54].

The first theories developed to study B-Z transitions treated highly simplified cases in which a transition could only occur at a single uniformly Z-susceptible site [16], [18]–[20], [25]. An extension of this approach has been developed, which uses a thermodynamic model to calculate the propensity of an individual segment, extracted from a genomic sequence, to form Z-DNA when placed in a Z-resistant background [22], [55]. However, a base composition-dependent statistical mechanical model is required to calculate the competitive B-Z transition behavior of kilobase length DNA sequences. We have recently implemented the first algorithm, called SIBZ, that performs this type of analysis [31]. The SIBZ algorithm uses the same basic computational strategy as SIDD, but substantial modifications were needed to treat the B-Z transition. The results of SIBZ agree well with experimental measurements of the onset of transition as a function of superhelicity [5], [52], as well as experimental determinations of the locations where the superhelical B-Z transition occurs within genomic DNA sequences [49]–[51].

In this paper we develop the first algorithm that evaluates the statistical mechanical equilibrium behavior of a negatively supercoiled DNA molecule of kilobase length and specified sequence that is susceptible to multiple types of conformational transitions. Our method calculates separate transition profiles (i.e. the probability of transition of each base pair in the sequence) for each type of competing transformation. It also can calculate ensemble averages of other important parameters, including the number of transformed base pairs of each type, the number of regions experiencing each type of transition, the overall probability of transition to each type of secondary structure, and the probabilities of different types of transitions occurring simultaneously. The algorithm we develop to handle multiple competing transitions is based on and generalizes the numerical strategy used in SIDD for approximating the exact partition function. Although it necessarily makes some approximations due to the computational limitations of exactly evaluating the partition function, the SIDD-based approach has been demonstrated to provide accurate results in reasonable computational times.

In principle the method we present can be used to analyze competitions among any number of different types of transitions. In practice, however, to make quantitative predictions one must know both the geometry of the relevant secondary structures and the base pair-specific energetics of transitions to those conformations. Here we implement the model for a three state system in which each base pair can occur in the B-form, which is regarded as the ground state, or in either of two alternate conformations. We explicitly analyze the competition between superhelical strand separation and B-Z transitions since their energy parameters are known at comparable temperatures and ionic conditions. Although the energetics governing these two transitions have the same orders of magnitude in the physiological temperature range, denaturation is more temperature dependent while B-Z transition causes greater relaxation. Our analysis shows that, because of these properties, the competition between these two types of transitions is quite complex, involving the interplay between base composition effects, imposed superhelical density, and environmental conditions. We call the new algorithm BDZ*trans*, for *B*-form to the *D*enatured and/or *Z*-form transitions.

## Methods

Constraining a DNA molecule either into a closed circle or a topological loop fixes its linking number 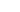, the number of times either strand links through the closed circle formed by the other strand. When the resulting topological domain is relaxed under physiological conditions, the molecule is entirely B-form with average twist rate of 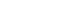 turns per base pair (bp). The linking number of the relaxed state of a domain containing 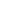 base pairs is given by 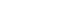.

A molecule that is negatively superhelical has linking number  that is smaller than its relaxed value, so its linking difference 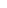 (also called its superhelicity) is 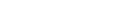. The superhelix density 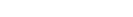 normalizes the level of superhelicity relative to the length 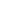 of the DNA experiencing it.

Superhelicity imposes torsional stresses on DNA. These stresses can be relieved by various deformations, including local conformational transitions to secondary structures having unstressed helical twist 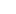 that is smaller than that of the B-form, 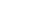. This includes a wide variety of DNA structures, such as strand separated DNA, Z-form, H-form, cruciform, and quadriplex DNA.

A statistical mechanical model is required in order to analyze the equilibrium transition behavior of a superhelical domain having a specified base sequence. At equilibrium the available conformational states are occupied according to their free energies. If a state 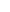 has energy 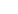, then at equilibrium it is occupied at a frequency proportional to 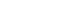, where 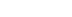. So the equilibrium probability of that state is 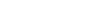, where the normalization factor 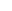 is the partition function of the system, given by 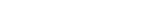. Once the resulting equilibrium distribution (also called the Boltzmann distribution) has been obtained, it is straightforward to compute ensemble averages of any parameter of interest [56]. If a parameter 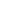 has value 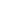 in a state 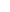, then its ensemble average value is
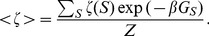
(1)


Analysis of the superhelical transition behavior of DNA requires knowledge of the structure of each alternate conformation, and of the free energies associated with transitions from the B-form to that conformation. The energy required to transform each base pair must be known, including its dependence on base sequence, under the conditions of temperature and ionic strength assumed in the calculations. In addition, there is a substantial nucleation energy for transitions to alternate DNA structures, which may be regarded as the energy needed to form junctions between that structure and its neighboring structures. Although this may vary according to the neighboring structure that it abuts, in practice this is usually B-form DNA. In cases where the alternate structure is flexible, such as strand separated DNA, significant twist can be absorbed by additional winding of that structure. This requires free energy that is modeled by an elastic Hooke's law as a quadratic function of the twist density. This term is not required for structures that are approximately as stiff as B-DNA, such as Z-DNA, or for structures that cannot undergo interstrand twisting, such as cruciforms. Finally, there is a free energy associated with the residual superhelicity 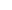, which is the superhelicity not absorbed by the changes of twist consequent on transition.

Suppose that in a state 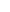 there are 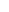 regions of alternate structure. Different regions may be in different alternate conformations. Let the 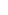-th such region consist of 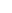 base pairs in an alternate conformation that has unstressed helicity 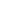. Its formation relaxes an amount of helicity given by 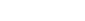 turns/bp. If this alternate structure is flexible, let each base pair be twisted by 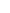 (radians/bp) away from its unstressed structure. If it is not flexible then 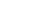. Also, suppose that the twist (in turns) of each junction at the 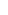-th region is 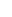. Then the residual superhelicity 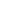 of the domain, which is the amount of superhelicity remaining to stress the molecule after all changes of twist due to transformations to the alternate conformations have occurred, is given by
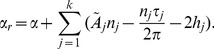
(2)The first term 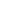 in this equation is the linking difference imposed on the molecule. The free energy associated with superhelicity has been measured to be quadratic [57].

### Theory of Competing Superhelical Transitions

Consider a topological domain consisting of 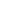 base pairs that is susceptible to 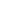 types of conformational transitions, 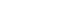. Suppose that a state 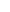 of this domain contains 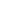 specific base pairs in conformation 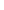. The total free energy of the state 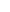 is given by
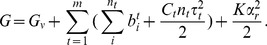
(3)The first term in this expression is the total nucleation energy 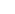 associated to this state. Suppose that there are 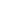 junctions between the B-form and the 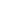-th alternate conformation, each of which has energy 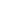. Also assume that there are 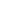 junctions between the alternate conformations 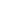 and 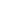, 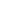, each with energy 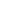. Then the total nucleation energy 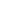 associated with this arrangement is
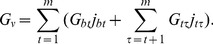
(4)


The second term in Eq. (3) sums the transition energies 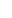 of the 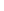-th base pair to be transformed to conformation 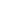 over the regions of transition. This transition energy varies with the type of transition the base pair experiences, the identity of the base pair (and sometimes also the identities of its neighbors), temperature, and ionic conditions. This term is summed over the number 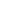 of base pairs involved in each transition.

The third term in Eq. (3) is the Hooke's law torsional energy associated to the twisting of alternate conformation 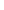. The parameter 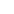 is the torsional stiffness coefficient of conformation 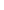, and 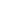 is its helical twist rate away from its relaxed conformation, measured in rad/bp. This term is required for strand separation, where the necessary parameter values have been determined [29]. However, it is not needed for transitions to other alternate conformations, such as the B-Z transition, whose twisting (along with that of the B-form DNA) is regarded as being incorporated into the residual superhelicity 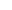. The free energy for conformations such as cruciforms, in which the two strands are physically separated in space and do not twist around each other, also does not contain this term.

More generally the helical twist could be considered to fluctuate independently for each transformed base pair. This has been done in a formally exact analysis of superhelical strand separation [30]. However, no significant difference was seen between the results of analyses that allowed independent twists, and those where all denatured base pairs were assumed to have the same twist 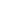, as in Eq. (3). Therefore, in this analysis we choose the latter strategy.

The last term in Eq. (3) is the quadratic energy associated with the residual superhelicity 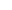, as defined in Eq. (2). The values of the constant 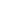 and the other energy parameters are discussed below for the specific transitions modeled there.

The expressions presented in this section can be applied to analyze molecules in which any number of superhelically driven transitions compete, provided the helical twist rates and transition energies of the alternate structures are known.

### The General Algorithm

Several computational strategies have been developed to analyze superhelically driven transitions in genomic DNA. Historically, the first fully developed algorithm focused on strand separation, since this is the only transition known to be required for essential biological processes such as the initiation of transcription and of replication. Although an exact theoretical method has been implemented that is capable of computing transition probabilities of individual base pairs in kilobase-scale genomic sequences, it proved too computationally cumbersome for widespread use [30]. However, an alternate strategy has been developed, called SIDD, that performs accurate and efficient approximate calculations. The initial SIDD algorithm focused on the superhelical strand separation transition [32]. This approach subsequently was modified into the SIBZ algorithm in order to treat superhelical B-Z transitions [31].

In this paper we further develop this computational strategy to enable efficient calculations of the equilibrium properties of superhelical molecules that are susceptible to multiple types of competing transitions. Although we focus specifically on the competition between denaturation and B-Z transitions in kilobase length superhelically constrained DNA sequences, in principle this approach can be applied to any number of different transitions.

The basic strategy of the algorithm is first to determine the lowest energy state of the system 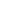. Then a threshold 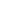 is set, and all states having energies less than 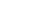 are found and included in the analysis [28], [32]. The number of states that are included, and hence the execution time, increases with 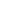, whose value must be chosen to suit the conditions assumed in the calculation and the level of accuracy desired.

Extensive calculations using both SIDD and SIBZ have shown that this approach has an attractive combination of efficiency and accuracy. Comparisons of the SIDD results with those from an exact method show that this approximate approach has an accuracy of at least four significant digits in all calculated parameters at physiologically attained superhelicities when a threshold 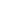 is chosen between 10 and 12 kcal/mol [32]. This accuracy is more than sufficient for comparison with experimental data. Although states having energies above the threshold are not explicitly included, a density of states technique has been developed that can approximately correct some calculated parameters for the cumulative effect of these high energy states [28]. Such corrections are beyond the fourth decimal of accuracy and hence are rarely needed in practice.

Calculations on 5 kb segments under standard conditions take on average about 10 seconds on one Opteron processor, although some segments can require up to 5 minutes or even longer. The difference in execution times strongly depends on the base composition of the sequence. For example, when there is a dominant transition region with a low energy cost, transformed states that do not include this region have a much higher energy. Therefore, relatively few states are found in the energy range determined by the threshold, resulting in a quick execution time. However, if no dominant transition regions are present, many states will have comparable energies, requiring a longer run time.

The free energy associated to each state of our competitive system, shown in Eq. (3), is comprised of two parts. The first two terms in this equation assign nucleation and base-dependent transition energies to the set of transformed base pairs in each state. These energies only depend on which base pairs are transformed and the alternate structures that they assume. As their domains are discrete sets, they are collectively referred to as the discrete part of the energy expression. Once the secondary structures of all base pairs have been specified, it remains to partition the balance of the superhelical deformation between residual superhelicity and twisting of the denatured regions. Since this partitioning can be done in a continuous manner, this term is described as the continuous part of the energy. It contains the energy terms arising from the superhelical constraint and from the twist. This separation of the energy expression into discrete and continuous parts facilitates finding the minimum energy state, as well as the states that satisfy the threshold condition, in a computationally efficient way [32].

We first calculate the energy associated to the discrete states, the nucleation energy given in Eq. (4) and the base-dependent transition energies 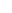. We consider segments of length 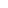 along the molecule. These segments are regarded as being susceptible to any type of transition, as long as they meet the sequence requirements for that alternate conformation. This is done for values of 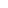 up to a limit 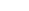. The value of 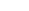 is chosen so that all states with longer runs of transition of any type will have energies higher than the threshold at physically reasonable values of the superhelix density 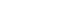. In addition, some transition types may have a lower limit on the segment length 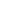.

Consider a circular molecule 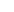 base pairs long. (We discuss linear molecules below.) In this molecule there are 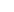 different segments of each length 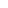, 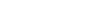, one starting at each base location. For simplicity, each segment is assumed to border B-form DNA on both sides. The total transition energy of each segment is
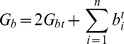
(5)The free energies found this way are sorted according to increasing energy into separate arrays for each transition type. The rows of these arrays are indexed by the length 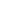 of the transformed segment. Each array has 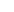 columns, equal to the length of the sequence being analyzed. In these arrays the first position of each transformed segment is stored along with its energy, as this information is required later in the calculation. Since the discrete components of the state energy are additive for multiple run states, these sorted arrays are also used to determine the discrete energies of states in which more than one run of transition is present.

To consider the one-run states of the system, we add the appropriate quadratic free energy associated with the residual superhelicity 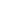 to each entry in the 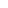-th row of all the arrays containing the discrete energies. This is done for each type of transition. (The manner in which the torsional deformation energy is treated in the strand separation transition is described in the next section.) The resulting energy values remain sorted within their rows, which enables an efficient search to be conducted for the lowest energy state among the untransformed or 1-run states. This lowest energy is taken as the initial value of 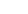. We next find all states whose energies satisfy 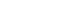, as described below. If in this process a multirun state is discovered whose energy is less than the current value of the minimum energy, then 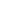 is assigned this lower value, which is used in the subsequent calculation. In practice this reassignment only occurs for a small fraction of sequences analyzed, and only when analyzed at extreme negative superhelicities. However, when it occurs more states are included than the final threshold cutoff condition requires, giving a correspondingly (very slightly) more accurate approximation.

For multiple run states, the procedure followed is similar to that described above for one run states. For each number of runs, the algorithm considers all 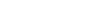 transition types, and the total number of runs 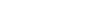, where 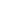 is the maximum number of runs considered. In general, each transition type includes a high initiation cost for each additional run. When the number of runs becomes large enough, all such states will have energies that exceed the threshold, and hence will not be included in the analysis. For this reason it is appropriate to impose a limit on the total number of runs that are considered. This is done by calculating whether any state with a given number of runs could satisfy the energy threshold condition by assuming that all transforming base pairs have the lowest possible transition energies, so the transition becomes isoenergetic. If it is found that such a state could satisfy the threshold condition, then a search of states with that number of runs can be instituted.

The Boltzmann factors associated with each state are accumulated into arrays for each transition type that are indexed by the lengths of their participating segments and their positions within the sequence. The contributions to the partition function for each type of conformation are collected separately in order to calculate their individual probability profiles. For multiple run states in which more than one type of transition occurs, the information for each run is placed in the appropriate array according to its length, position, and transition type. Details of these procedures may be found elsewhere [32].

A variety of equilibrium properties of the transition may be calculated from the information that is collected in these arrays. This includes the probability that each base pair in the sequence is in a particular alternate conformation, the expected number of runs of each transition type, the probability of the state with no transition, and other attributes of interest.

### Denatured DNA vs Z-form DNA

We focus henceforth on analyzing the competition between denaturation and B-Z transitions in a superhelical plasmid 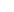 base pairs long and having any specified sequence. We first examine the residual superhelicity associated with this competition, given in Eq. (2), and then consider the state energy described in Eq. (3).

Strand separated DNA, being untwisted when unstressed, has 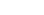 turns/bp. It follows that the transition of 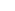 base pairs from B-form to the unstressed strand separated state involves a twist decrease of 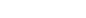 turns. Since single-stranded DNA is highly flexible, the two separated strands in a melted region are able interwind in order to further relieve supercoiling stresses. The amount of helical interwinding that occurs is denoted by 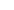 in radians/bp.

The helicity of Z-DNA is 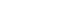 turns/bp, the minus sign indicating that it is twisted in the left-handed sense. So the decrease of helicity for each base pair experiencing this transition is , which is approximately 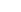 of untwisting per transformed base pair. Since the Z-form is torsionally stiff we do not consider its twist fluctuations separately, but rather regard them, together with those of the B-form regions, as part of the residual superhelicity. In addition, each B-Z junction requires an untwisting of 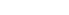 turns [21].

Since the Z-form is favored in G+C-rich regions and strand separation in A+T-rich regions, in practice they are unlikely to both be competitive at the same regions. In particular, junctions where strand separated DNA directly abuts Z-form DNA are unlikely to occur in low energy states under the conditions assumed below. In this case the nucleation energy of Eq. (4) can be written as
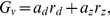
(6)where there are 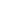 runs of conformation 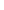 (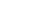). (A run is defined as a segment in which all base pairs are in the same alternate structure.) Here the nucleation energy of a single run of type 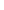 is 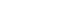, the cost of producing two junctions between B-DNA and that conformation.

We consider a state in which there are 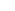 denatured base pairs in 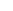 runs, and 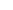 Z-form base pairs in 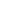 runs. Because the unit cell of Z-DNA is a dinucleotide, 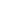 is an even number. Then the residual superhelicity 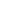 whose general form is given in Eq. (2) becomes
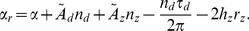
(7)


The total free energy associated to this state is given by
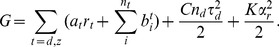
(8)The values of the various energy parameters found in this equation are discussed in the section below.

In describing a particular state one first specifies the conformation of each base pair in the sequence being analyzed. Here they may be either B-form, Z-form, or melted. This determines the numbers 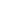 and 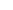 of transformed base pairs, and the numbers 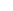 and 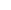 of runs for each transition. This fixes all the factors in Eq. (7) except for the residual superhelicity 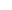 and the twist 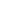 of the denatured regions. There is a continuum of ways to partition the balance of the topological constraint between 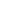 and 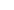 of the single stranded regions. In previous papers we have developed and evaluated a number alternative ways of treating this partitioning [28], [30]. We found that high accuracy can be achieved by minimizing the total free energy associated with these two quantities, which are the two terms on the right in Eq. (8), subject to the condition that the sum 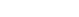 remains constant. This minimum occurs when
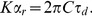
(9)Combining previously described terms and using this minimization condition in Eq. (8), we obtain the following expression for the free energy of a state when denaturation and B-Z transitions compete:
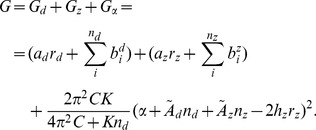
(10)


### Energy Parameters

In the present implementation we assume that strand separation is governed by copolymeric transition energies. That is, every 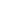 or 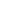 base pair is assigned the same separation free energy 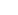, while every 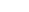 or 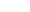 base pair is given separation free energy 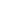. Nearest neighbor energetics have been measured for strand separation under various environmental conditions [58]–[60], and their use has been implemented as an option in the SIDD algorithm [61]. Although these can easily be incorporated into the present analysis, we choose to use the computationally slightly faster copolymeric energetics, since little practical difference has been seen between the results found using these two approaches.

The free energy of strand separation depends on temperature according to the relationship
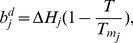
(11)where 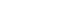 for A or T bases and for C or G bases, respectively. The enthalpy 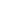 of this transition has been measured to be 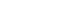 kcal/mol and 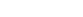 kcal/mol [29]. The entropy term in this equation is related to the transition temperature 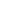, which is the temperature at which the transition energy 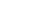. In turn, 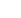 varies with ionic strength 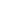 according to
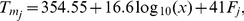
(12)where 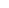 is the salt concentration in molar units, the temperature is in degrees Kelvin, 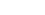, and 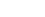.

The remaining energy parameters found in Eq. (10) that involve strand separation have been well evaluated at salt concentration 0.01 M and temperature 310 K, where the most sensitive experiments to analyze superhelical strand separation were conducted [9]. Analysis of these experimental results determined the torsional stiffness coefficient to be 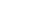 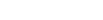, and the nucleation energy to be 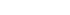 kcal/mol [62]. We assume no temperature dependence for these parameters. The superhelical energy parameter is 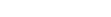, where 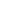 is the gas constant, 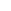 is the temperature, and 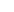 is the number of base pairs in the sequence being analyzed [63], [64].

The unit cell of Z-DNA is a dinucleotide (i.e. two neighboring base pairs), with one in the *anti* and the other in the *syn* configuration. Therefore, we henceforth regard the B-Z transition energy 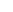 as referring to the energy of forming a unit cell of Z-DNA, hence associated to two base pairs. In addition, there is an energy cost required when two neighboring dinucleotide repeat units break the *anti-syn* alternation, as happens for example in the (AS)(SA) arrangement. This Z-Z junction energy is denoted by 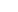 and is added to the total Z-forming free energy of states in which these junctions occur [31].

B-Z transition energetics have been determined for each of the ten possible dinucleotides. Most of these free energies were experimentally measured at room temperature and 0.1 M sodium concentration [21], [52]–[54], although some were estimated [22], [25]. The B-Z transition energies of all ten dinucleotide pairs and the corresponding Z-Z junction energies at these environmental conditions are given in [22], [31].

When the transition properties of uniformly Z-susceptible inserts within a pBR322-derived plasmid were determined from two-dimensional gel electrophoresis experiments, it was found that the B-Z transition behaved the same at 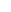
C as at 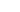
C [26]. This suggests that there is no significant temperature dependence of the B-Z transition, at least for the inserted sequence that was used. For this reason we assume that the dinucleotide, Z-Z junction, and the B-Z junction energies are all independent of temperature. If future measurements find differently, this assumption can easily be modified.

There is an intricate interplay between the superhelical B-Z and local denaturation transitions that arises from the different energy dependencies of these reactions. Transition to Z-form involves a greater change of twist than does strand separation, so it relieves more superhelical stress. This suggests that the B-Z transition will occur at less extreme superhelicities than strand separation at physiological temperatures, other influences remaining fixed. However, the transition free energy of strand separation is highly temperature dependent, whereas the B-Z transition appears to be approximately independent of temperature. In consequence, as temperature rises one expects a change of behavior in a superhelical molecule that is susceptible to both types of transitions. At relatively low temperatures one expects B-Z transitions to dominate, with strand separation occurring only at more extreme superhelicities after the low energy Z-susceptible regions have transformed. However, as the temperature rises the free energy cost of strand separation diminishes, so one expects this transition to become more competitive. The calculations we report below suggest that these energy effects can render the competition between these two types of superhelical transitions quite complex in practice.

### The BDZ*trans* Algorithm

In previous sections we summarized the general algorithmic strategy for treating multiple transition types. Here we describe how this algorithm is tailored specifically to model the competition between superhelical strand separation and Z-DNA formation.

The state energy has been separated into the discrete (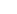 and 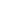) and continuous (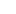) parts as described in Eq. (10). We first consider the energy associated to the discrete states. We regard each segment of length 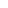 along the molecule to be susceptible both to strand separation and, when 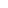 is even, to Z-form. In a circular molecule of length 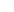 and longest segment 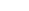 this produces a matrix of denaturation energies of dimension 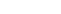, and a matrix of B-Z transition energies of dimension 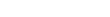. (Here the square brackets denote the greatest integer function.) We limit the minimum number of Z-forming dinucleotides in a single run to four, since shorter runs of Z-DNA have not been found experimentally [21]. We subtract three from the number of lengths considered because we do not include Z-runs comprised of 2, 4, or 6 dinucleotides.

As described in the previous section, we assign copolymeric energies to denatured regions according to their base sequences. It follows that the discrete energy associated with the denaturation of 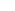 specific base pairs is
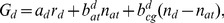
(13)where 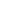 is the number of denatured 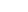 or 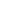 base pairs, so 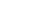 is the number of denatured 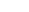 or 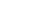 pairs, and 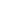 is the number of denatured regions present.

Next, we determine the energetics of transition to Z-form of 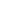 runs that together contain 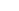 transformed dinucleotides, hence 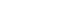 base pairs. First, we find the most energetically favorable *anti*/*syn* conformation according to its base sequence, and then we calculate the total energy by summing the energies of each Z-DNA dinucleotide and all the occurring Z-Z junctions. Details of this procedure are provided elsewhere [31]. The discrete B-Z transition free energy is given by
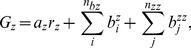
(14)where 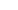 is the number of Z-Z junctions, and 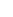 and 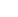 are the dinucleotide and junction energies, respectively.

The discrete energies are calculated from the above equations for single runs of transition of any length in the range 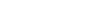. The free energies found this way are sorted according to increasing energy into arrays 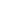 for denaturation and 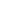 for the B-Z transition as described above. The rows of these arrays are indexed by the length of the transformed region, which is 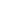 base pairs for denaturation and 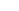 dinucleotides for the B-Z transition.

In each execution of the algorithm one initially fixes the imposed linking difference 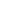 and the temperature 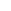. This determines the parameters associated with the continuous component 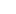 of the state free energy. This is the quadratic last term in Eq. (10), which varies with the numbers 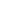 of melted bases and 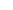 of Z-form bases, and the number of Z-runs 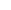: 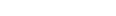. We note that 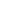 does not depend upon the positions of the runs of transition within the sequence. If there are 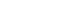 runs of transition in a state and 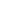 transformed base pairs in the 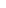-th run, then
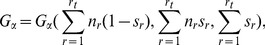
(15)where 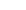
{0, 1} denote the denatured and the Z-form states, respectively.

For one-run states of the system, we add 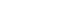 to each entry in the 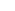-th row of the array 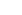 containing discrete energies for strand separation, and 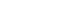 to the corresponding entries of the 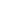 array. Since the nucleation energy is significant for both denaturation and B-Z transitions (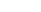 kcal/mol in both cases), a limit on the total number of runs considered may be imposed. In SIDD analyses with threshold 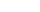 kcal/mol it was found that states with more than three runs were never found under reasonable conditions [32]. For the SIBZ algorithm it was determined that states with more than four runs would generally not occur [31]. Therefore we impose a limit of four runs in the present algorithm.

For each number of runs, the algorithm is iterated for 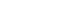, where 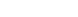 when a maximum of four simultaneous runs is allowed. This arrangement assures that there are three types of two-run states (two melted regions, two Z-regions, or one melted and one Z-region). Similarly, there are four types of three-run states, and five types of four-run states. The energy associated with a state is calculated by adding the appropriate energies from the discrete arrays to the total superhelical energy 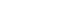 for each number of runs of the appropriate types. For example, the energy of a two-run state where 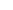 bases are strand separated and 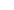 bases are in Z-form is 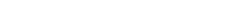, where 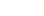 and 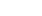 depend on the base sequence of the runs involved. Whenever the total energy associated to a state satisfies 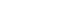, its Boltzmann factor is calculated and added to the appropriate arrays as described above.

We analyze linear molecules by connecting their ends with an inserted sequence, and then treating them as circular. To fully isolate one end from the other, the insert must be chosen so it is energetically highly disfavored to undergo any transition. For the B-Z and denaturation transitions this is achieved by inserting a 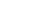 segment between the ends. Since this run of G's is unlikely to either melt or form Z-DNA, its insertion prevents any artificial correlation between the two ends from arising, hence correctly simulates a linear sequence. The linking difference that corresponds to the specified superhelix density is imposed on the resulting molecule. The algorithm only reports the results for the actual sequence, and disregards those from the insert.

### Computational Accuracy of the BDZ*trans* Algorithm

In order to assess the computational accuracy of the BDZ*trans* algorithm we compare its results to those from an exact analysis of a simplified situation in which competition is limited to two homopolymeric sites. Specifically, we consider a 5 kb plasmid in which there is one uniformly Z-susceptible region and a second region, at a distance from the first, that is uniformly susceptible to denaturation. The A+T-rich, easily melted segment has length 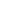, while the Z-susceptible site is a dinucleotide 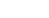 repeat containing 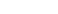 base pairs. By uniform susceptibility we mean that both transitions are homopolymeric: all dinucleotides in the Z-susceptible segment have the same transition energy 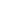, and all base pairs in the denaturation-susceptible region have the same transition energy 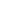. All other parts of the plasmid are regarded as being unable to undergo any form of transition, so the competition is exclusively between these two sites. In our simulations this is achieved by giving high transition energy values to all base pairs that are not in these regions. This example approximately corresponds to an experimental situation where a highly Z-susceptible sequence, such as 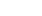, is inserted into a plasmid that contains a dominating SIDD site, such as the 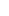-lactamase terminator in the pBR322 plasmid.

This case is analytically solvable by standard procedures that have been presented and applied elsewhere [16], [26]. Here we also solve it using the BDZ*trans* algorithm, and compare the results. In the BDZ*trans* analysis we use an energy threshold of 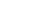 kcal/mol and consider only states with four or fewer runs of transition. Calculations were performed using both methods over a range of superhelical densities and temperatures for various combinations of segment lengths 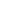 and 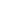.

The analytic calculation allows the A+T-rich segment only to melt, and the 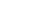 segment only to assume the Z-form. However, the BDZ*trans* algorithm allows both regions to undergo either type of transition. In all situations where these two segments either are untransformed or experience their expected transitions, we find that the results from the two methods agree exactly up to the accuracy of double precision. (Data not shown.) Having established the high computational accuracy achieved by the BDZ*trans* algorithm, we now can use it to analyze other situations, where exact calculations are not possible.

## Results

The BDZ*trans* algorithm has been applied to analyze several situations, the results of which are reported here. First, we consider a simplified case where a plasmid contains two regions that are uniformly susceptible to superhelical transitions, one to strand separation and the other to the B-Z transition. Second, we perform an analysis of the only experimental data presently available regarding the competition between B-Z transitions and strand separation in superhelical plasmids. We show that the predictions of BDZ*trans* agree closely with this experimental data when energetics that are appropriate to the buffer conditions of the experiment are used. Third, we analyze the competition between strand separation and B-Z transitions in the pBR322 plasmid. Specifically, we demonstrate that there is a strong temperature dependence to this competitive behavior. Fourth, we analyze the superhelical competition between B-Z transitions and denaturation in the control regions of the *c-myc* oncogene. Both transitions have been shown to occur when this gene is transcribing, and have been proposed to regulate its expression. However, to date little is known regarding the competitive interactions between these transitions. Lastly, we apply BDZ*trans* to study transition behavior around genomic sites that regulate transcription. We compare the patterns of transition found in eukaryotic genomes around transcription start sites (TSS) with those that occur around the sites where the transcript terminates. We also compare the patterns around the TSS that are found in eukaryotes (human and mouse) with those found in a prokaryote (*E. coli*), and with those that occur in a class of pseudogenes that are not transcribed.

### The Competition Between Two Regions

We first analyze the competition between two regions in an otherwise transition-resistant background. The specific problems addressed here were chosen to eluciate the complexities that can arise in superhelical competitions between strand separation and B-Z transitions, even in simplified situations. The intricacies of these interactions result primarily from three factors. First, as shown in Eq. (11) and Eq. (12), strand separation is strongly temperature dependent, with the transition temperatures of specific regions depending both on base composition and on ionic strength. In contrast, the B-Z transition appears to be essentially independent of temperature [26]. One anticipates that this will cause significant variations with temperature of the competition between these two types of superhelical transitions. Second, the B-Z transition relaxes substantially more superhelical stress per transforming base pair than does strand separation. Lastly, the relative lengths of susceptible regions also can strongly influence their competitions [18].

We first consider the case where the Z-susceptible insert is a 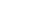 sequence placed at position 1000 in a 5 kb plasmid, and the denaturation-susceptible region is 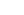 at location 3000. The temperature is set at 300 K, and the superhelix density 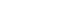 is allowed to vary. We only consider negative superhelix densities 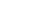, although the results are presented in graphs as a function of 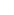. Since 300 K is substantially lower than the transition temperature for poly-A (see Eq. (12)), these conditions should favor the B-Z transition. However, as the A-rich region is long, its transition will produce more relaxation than will the short Z-forming region. We use BDZ*trans* to calculate the probability of transition of each base pair in each susceptible region. We then average these values over the lengths of the regions involved to find the average probability of melting of base pairs in the A-segment, and of Z-formation in the CG-region.

**Figure 1:**
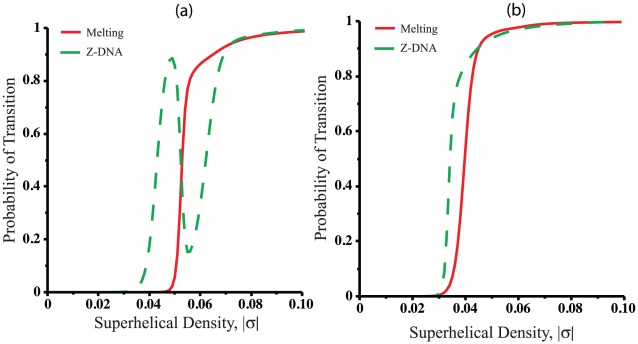
The average probabilities of transition calculated as functions of negative superhelicity (here plotted as 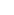) for a 5 kb plasmid containing a single denaturation-susceptible site and a single Z-susceptible site in an otherwise transition-resistant background. The analysis is done for the melting region 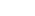 and the Z-susceptible segment 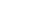 st 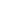 = 300 K using (a) BDZ*trans* (b) SIDD for denaturation and SIBZ for Z-DNA.

The results of these calculations are shown in Fig. 1(a), which graphs the average probability of each type of transition in the corresponding segment as a function of superhelicity. When 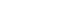 there is not enough superhelical stress to drive any transition. As the superhelix density becomes progressively more extreme, B-Z transition in the 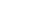 segment occurs first and dominates in the range 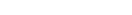. However, since the Z-susceptible region contains ten base pairs, it can only relax less than two superhelical turns. Although denaturation relaxes less stress per transformed base pair, in this sequence the A-rich segment is much longer than the Z-susceptible segment, and hence can relax substantially more superhelicity. In the range of 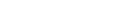 there is a coordinated reversion of the Z-forming region back to B-form, coupled to denaturation of the A-rich segment. In this range it is energetically too costly for both transitions to occur, so transformation of the longer meltable region becomes favored because it provides more relaxation. When 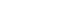 the 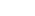 region is essentially fully melted, and additional stress induces the B-Z transition. Around 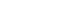 both segments are essentially completely transformed. Similar coupled transition-reversion events have been noted for other competitions, including those between two Z-susceptible regions [24], [31], between two cruciform extrusions, and between cruciform extrusion at one site and B-Z transitions at another [18].

In Fig. 1(b) we show the results for the same system obtained when it is analyzed using the two-state algorithms, SIDD for denaturation and SIBZ for the B-Z transition. One sees that disregarding the competition between different types of transitions can result in an entirely incorrect representation of the transition behavior of the plasmid. First, the onset of the melting transition occurs at a lower value of 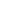 in Fig. 1(b), since in SIDD denaturation is not competing with the B-Z transition, which is first to transform in the competitive situation, as shown in Fig. 1(a). Further, the reversion of the B-Z transition apparent in Fig. 1(a) is not captured here, because the competition between that transition and melting is not considered. Using the individual algorithms separately wrongly predicts a B-Z transition with probability close to one for 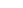
−0.055, whereas including the competition with denaturation shows this probability actually to be below 0.2.

**Figure 2:**
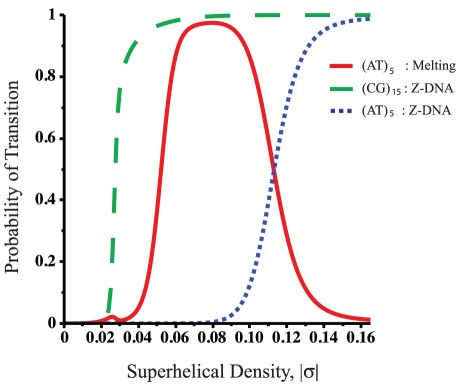
The average probabilities of transition calculated as functions of negative superhelicity for a 5 kb plasmid containing a single denaturation-susceptible site and a single Z-susceptible site in an otherwise transition-resistant background. The average probabilities are shown of 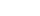 either melting (solid) or forming Z-DNA (dotted), and of B-Z transition at 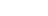 (dashed) at temperature 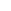 = 340 K.

Next, we analyze the reverse situation, in which a short 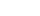 melting region competes with a longer 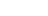 Z-susceptible region. These calculations were performed at a temperature of 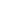 = 340 K, which is higher than the transition temperature of 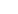 = 322 K for A+T-rich DNA at 0.01 M salt concentration. The average transition probabilities calculated for this situation are plotted as functions of 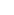 in Fig. 2. Although the high temperature should favor denaturation, the high nucleation energy of denaturation keeps the 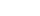 region in the B-form state when the molecule is relaxed at this temperature. The onset of transition under these conditions occurs around 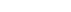. Since the Z-susceptible site is much longer and hence can relax more superhelicity, B-Z transition is the first to occur. The short AT-region can only relax about 1.5 turns of superhelicity while B-Z transition, although energetically more expensive, can relieve much more stress. This greater stress relief favors the latter transition, even at this high temperature. At 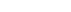 the B-Z transition becomes essentially complete, and denaturation starts to occur as a second transition. In the range 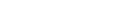 both segments are completely transformed. However, at extreme superhelicities of 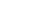, the melting probability of the 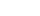 segment is seen to fall gradually back to zero. At this level of supercoiling BDZ*trans* finds that this segment transitions from the denatured state to the Z-form. This behavior can be understood by comparing the energies required for the AT-insert either to melt or to form Z-DNA, assuming that the entire 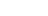 segment is already in Z-form. By equating these two free energies, it is straightforward to obtain the critical superhelix density 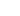 at which the 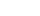 insert begins to favor the B-Z transition over denaturation. For this sequence at T = 340 K we find 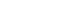. The value for 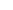 agrees exactly with the result obtained by BDZ*trans* for 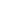 where the two probabilities of the 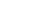 segment are equal, which occurs at the intersection of their two curves in Fig. 2. This behavior also could not be predicted from separate analyses of each type of transition.

These examples illustrate some of the complexities that can occur in multi-state superhelical transitions, even in artificially simple situations. In particular, the susceptibility of a region within a sequence to undergo a certain transition is not always a simple function of its base composition. Although at high temperatures it generally it takes less energy to melt an A+T-rich region than to transform it to the Z-helix, the results presented in Fig. 2 shows that under certain circumstances the opposite behavior can happen at equilibrium, even well above the melting transition temperature where one might imagine that denaturation would dominate. These examples also show the importance of competing together all transition-susceptible sites in the sequence, rather than simply analyzing the propensity of each individual region to transform independent of the rest of the domain.

### Comparisons with Experiment

To date only one experimental investigation has been performed of the competition between melting and the B-Z transition in supercoiled DNA [26]. The pAT153 plasmid used in those experiments is a derivative of pBR322 that contains an A+T-rich, easily meltable 105 bp region at its 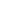-lactamase gene terminator. Two other plasmids were constructed by inserting a Z-susceptible sequence into pAT153 in order to observed the competition between strand separation and the B-Z transition in a situation where the regions involved do not abut. The pCG8/vec plasmid was constructed by inserting the highly Z-susceptible 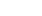 sequence, and the pTG12/vec plasmid was constructed by inserting 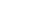. This insert is also susceptible to B-Z transition, although it requires approximately twice the energy per dinucleotide to transform as does 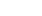.

Each plasmid was subjected to two-dimensional gel electrophoresis to determine its transition behavior over a wide range of linking differences. The amount of residual superhelicity 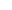 present at each linking difference can be measured directly from the 2-D gel data, as described elsewhere [65]. From this information the extent of transition-induced relaxation can be found as 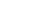. We analyzed the transition behavior of each of these three plasmids directly from the original gel images, which were kindly provided by the experimental investigators [26].

To compare these experimental results with theory we used the BDZ*trans* algorithm to calculate the equilibrium transition behavior of each complete plasmid over the experimental range of linking differences. We consider superhelical competition between all base pairs in each plasmid, rather than isolating the competition between the transition susceptible sites. By inserting the condition from Eq. (9) into Eq. (7), we obtain the following expression for the ensemble average value 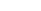 of the residual superhelicity when strand separation and B-Z transitions are competing:
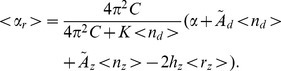
(16)Here the expressions in angled brackets are ensemble averages of the bracketed parameters, which are calculated using our numerical method. The relaxation experienced by a topoisomer due to transitions is given by 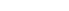. This quantity can be compared to the extent of relaxation experienced by the experimental plasmids, which is determined from the 2-D gels.

A single transition was observed experimentally in the pAT153 plasmid, which contains the A+T-rich region found in pBR322, but no Z-susceptible insert. This transition was reported to be highly temperature dependent, indicating that it is strand separation [26]. In contrast, two transitions were observed as negative superhelicity was increased in the pCG8/vec plasmid at 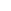 = 305 K. The first transition, at the less extreme superhelicity, was suggested to be a B-Z transition both by chemical probing, and by the degree of unwinding it exhibited. This transition was found to be essentially independent of temperature, behaving the same at 281 K as at 305 K. This further confirmed its B-Z character, as strand separation is well known to be highly temperature dependent. The nature of the second transition in this plasmid was not determined experimentally. However, it was assumed to be denaturation, primarily because it behaved in a qualitatively similar manner to the transition observed in pAT153. The pTG12/vec plasmid also showed two transitions, which were delayed in linking difference relative to those seen in pCG8/vec.

The 2-D gel experiments were performed in TBE/2 buffer, which contained 45 mM tris borate and 0.5 mM EDTA at pH 8.3. Unfortunately, the energetics of denaturation and of the B-Z transition have not previously been determined under these conditions. In particular, the energetics for strand separation described previously were determined at pH 7.0 [9], and no correction for a higher pH is known. Since this difference in pH level constitutes a 20-fold decrease in the 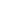 counterion concentration, it is reasonable to suppose that it could affect the energetics of melting, which are known to vary with the concentrations of larger monovalent counterions.

When we ran the BDZ*trans* algorithm on the pAT153, CG8/vec, and TG12/vec plasmids using the energy values described in the “Energy Parameters” section, we found that their qualitative experimental behaviors were correctly depicted by the numerical model. However, it was apparent that the transition energy parameter values used were too large, since the transition behavior predicted by BDZ*trans* was consistently shifted to more extreme superhelicities than were observed experimentally. Therefore, our first task in comparing numerical calculations with experiments was to determine the transition energetics appropriate to the experimental conditions.

We did this in the following manner. First, we used the SIDD algorithm, which considers only strand separation, to fit the experimental data on the melting transition in pAT153. To determine the relaxation as described above, we set the parameters 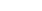 and 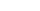 to zero in Eq. (16), since the B-Z transforming insert is not present in this plasmid. When performing these fits to the data, we chose to vary only the transition energetics per base pair and to keep all other parameters fixed. The best fit with experiment was achieved when the sequence averaged transition energetics of the easily melted region are 0.45 kcal/mole per bp. This is about 0.3 kcal/mole/bp smaller than the value derived from the information in the “Energy Parameters” section, which pertain under other experimental conditions. It is well known that the melting energy of DNA decreases as salt concentration is lowered, as is shown in Eq. (12). The present analysis suggests that a similar decrease may also occur when the counterion is 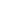.

Next, we used the SIBZ algorithm, which considers only the B-Z transition, to analyze the first transition in the pCG8/vec plasmid. In this case we set 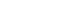 in Eq. (16), since there is no denaturation in this regime. We find that the best fitting B-Z transition energetics for the CG dinucleotide is approximately 0.1 kcal/mole/bp lower than the values found in [22], [31]. The same analysis of the first transition in the pTG12/vec plasmid gives a similar result for the TG dinucleotide. We note that these results are in qualitative agreement with those found by the authors of the experimental paper [26], who used a different and rather simpler method of analysis.

**Figure 3:**
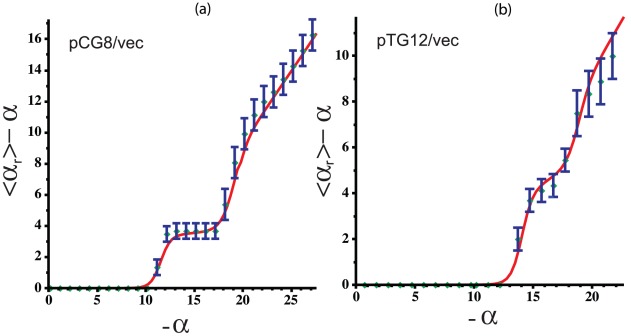
Comparison of the BDZ*trans* algorithm results (solid line) with experimental data (points) derived from 2-D gels [26] for the two plasmids shown. The horizontal axis plots the negative superhelicity while the vertical axis shows the relaxation provided by transitions. Both quantities are in units of turns. The first transition in each plasmid is B-Z, and the second is denaturation. (a) The transition behavior is plotted of the pCG8/vec plasmid, which contains a low energy Z-susceptible 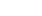 insert; (b) The transition behavior is shown of the pTG12/vec plasmid, which contains a higher energy Z-susceptible 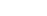 insert. The error bars on the experimental data points arise from the uncertainties inherent in the gel images and in the process of reading them.

Finally, we used the BDZ*trans* algorithm to analyze the full competition between strand separation and B-Z transitions in the pCG8/vec and pTG12/vec plasmid sequences. This was done at T = 305 K using the fitted energy parameter values found above for both transitions. The BDZ*trans* results are plotted as solid lines in Fig. 3(a) and (b), while the dots with error bars represent experimental data. The horizontal axis shows the imposed superhelicity 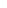, and the vertical axis plots the relaxation 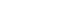. We find close agreement in both cases between the results of BDZ*trans* and the experimental data. This accord shows that, given the correct energy parameters, the BDZ*trans* algorithm captures the competition between two alternate structural transitions in a quantitatively accurate manner.

### Analysis of the pBR322 DNA Sequence

Both experiments and SIDD analysis have shown that the superhelical pBR322 plasmid contains a dominant melting region that is about 105 bp long and coincides with the 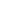-lactamase gene terminator [9], [30]. Antibody binding experiments and SIBZ analysis of this plasmid has shown that it also contains several short Z-forming segments, the longest of which consists of 14 bp [31], [66]. Here we use the BDZ*trans* algorithm to analyze how these two transitions compete under various conditions. Specifically, we calculate the probability of each transition occurring anywhere in the plasmid. This probability is defined as 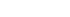, where 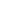 = 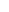, 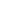, and 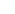 is the sum of all the Boltzmann factors for all states in which at least one region is in conformation 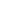. The probability 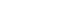 that both types of transition occur in the same molecule also is calculated. We analyze the transition behavior of this plasmid at base pair resolution by also calculating the probabilities of each base being in either alternate conformation at equilibrium. The transition profiles show the graphs of these probabilities as functions of position.

**Figure 4:**
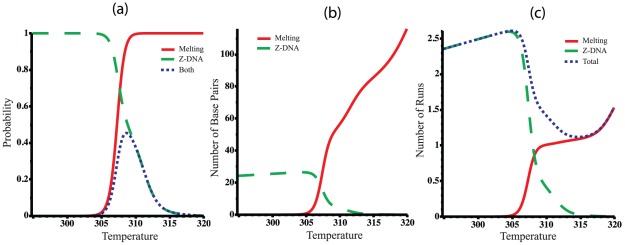
Various thermodynamic equilibrium properties of competing denaturation and B-Z transitions in the pBR322 sequence are shown as functions of temperature. All calculations were performed at 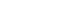: (a) The probability of each transition 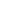, 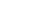, are shown, as is the probability 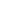 of both transitions occurring simultaneously; (b) the average number of transformed base pairs 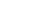 is shown for each transition type; (c) the average number of runs 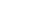 are plotted for each transition type. The average total number of runs 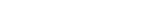 also is shown.

First, we used BDZ*trans* to analyze the pBR322 plasmid sequence at superhelix density 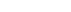 and various temperatures. The results are shown in Fig. 4(a), which plots the probabilities 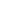 of each transition as a function of temperature. It is apparent that the B-Z transition dominates at low temperatures, while strand separation prevails at high temperatures. The probability 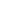 of both transitions occurring simultaneously also is shown. Although at 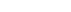 the value of 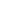 never exceeds 0.5 at any temperature, at more extreme superhelicities both transitions will occur simultaneously with high probability. We define the phenomenological competitive transition temperature 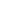 to be the temperature at which both transitions are equally probable. At this superhelix density 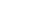 K.

Figs. 4(b) and 4(c) plot the average numbers of transformed base pairs and runs of transition, respectively, for the pBR322 plasmid as functions of temperature at 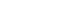. Separate curves are shown for each transition type, and the total number of runs for both transitions is also given. The transition behavior seen in these graphs arises from the fact that the Z-susceptible regions in the pBR322 plasmid are several but short, while the region most susceptible to strand separation is about 105 bp long. At low temperatures and 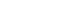 the B-Z transition is seen to dominate over strand separation. In this regime, although multiple sites are in Z-form, they together comprise only 25 to 30 base pairs on average. The difference in the numbers of base pairs undergoing each type of transition that is seen in Fig. 4(b) is also a consequence of the fact that the B-Z transition relieves substantially more superhelical stress per base pair than does strand separation.

As the temperature increases beyond 308 K strand separation comes to dominate, and the propensity to form Z-DNA falls back to zero. In this regime the number of denatured base pairs increases to large values. This behavior is a consequence of the strong temperature dependence of denaturation. With increasing temperature the energy required to denature a region goes down, so more of the imposed superhelicity is partitioned to this transition at equilibrium. Since the dominant destabilized site is long, all this melting can be accomodated within that site until the temperature reaches around 315 K, where the average number of runs start to exceed one, as shown in Fig. 4(c). At this point the first site is fully melted and a second site located near the promoter region of the 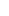-lactamase gene also starts to melt.

**Figure 5:**
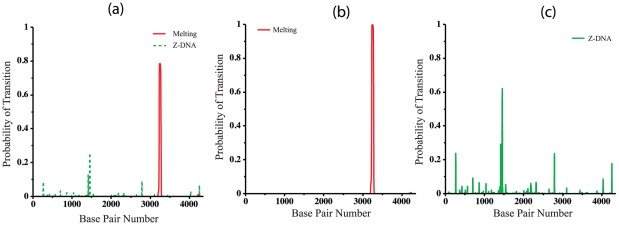
The transition profiles calculated using three different algorithms are shown for the pBR322 plasmid. These calculations are performed at 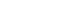 and 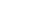 K using (a) BDZ*trans*, (b) SIDD, and (c) SIBZ.

Next, we compare the transition profiles calculated by BDZ*trans* for each type of transition with those calculated for denaturation alone by SIDD and for B-Z transitions alone by SIBZ. These three profiles are calculated at superhelix density 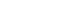 and 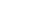 K, and are shown in Fig. 5. Comparison of these profiles shows that the sites that transform when the two transitions are competing coincide with the dominant sites that are predicted when each transition is treated alone. However, the probabilities of transition at these sites are significantly smaller when the two types of transition are allowed to compete. The probability of the dominant melting region drops from 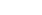 when calculated by SIDD to 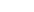 when calculated by BDZ*trans*. Similarly, the transition probability of the dominant Z-forming region changes from 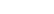 when only the B-Z transition is allowed, to 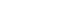 when both conformations compete. One sees that calculations in which only one alternate conformation is considered tend to overstate the transition probabilities relative to a more realistic analysis in which multiple transition types compete together.

Table 1 shows sample numerical results and technical information regarding these computations. The execution times reported here are for calculations performed on a MacBook Pro with dual Intel processors. The SIBZ algorithm is slowest, as the B-Z transition occurs at multiple runs (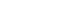) under these conditions. In consequence, SIBZ also includes the largest number of states. SIDD is fastest because melting occurs predominantly in single run states, so fewer states satisfy the threshold condition for this transition. When the full competition is analyzed using BDZ*trans*, the average number of runs of denaturation and of Z-formation are both smaller, so there are an average of 1.8 runs in this case. The total number of transformed base pairs also decreases for each transition. The execution time of BDZ*trans* is intermediate between those of SIDD and of SIBZ, as is the number of states it includes.

**Table 1:** Numerical results for pBR322 calculated at 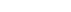 and T = 308 K using the SIDD, SIBZ, and BDZ*trans* algorithms.

Algorithm	time (min)	number of states	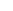	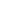	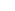	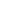
SIDD	0.067	2187531	1.0	-	53.5	-
SIBZ	0.85	452111726	-	2.7	-	27.5
BDZ*trans*	0.43	160664229	0.80	1.00	39.4	10.0

In all cases a threshold of 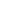
12 kcal/mol was used. Values are shown for the total execution time, the total number of included states, the average number of runs 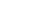, and the average number of transformed base pairs 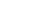.

These calculations show that the results from BDZ*trans* are qualitatively consistent with those from SIDD and SIBZ in that the competing transitions are largely limited to sites that dominate when each transition is considered alone. However, the probabilities of transition found by BDZ*trans* are not related in any simple way to those found by the other two algorithms. In particular, they are not weighted averages of the probabilities found by SIDD and SIBZ, and there is no direct way in which one could estimate the competitive behavior of these two transitions from the profiles found using the independent analyses. Rather, this behavior is determined by complex, globally coupled, non-linear interactions, and can only be assessed by a full analysis that simultaneously considers both types of competing transitions.

### Competing Superhelical Transitions at the Human *c-myc* Oncogene

Regulation of the *c-myc* oncogene has been intensively studied, in part because mutations involving this gene have been implicated in various cancers. Substantial evidence has been found suggesting that superhelical DNA structural transitions play roles in regulating *c-myc*. Its 5′ flank contains a SIDD site called FUSE, located 2 kb upstream from the promoters, three *Alu*I fragments containing Z-forming sites, and a potentially either G-quadriplex forming or H-forming site called the CT element around 1 kb upstream from the promoters [14]. Experiments have shown that each of these sites can be driven into their alternate structure by the superhelicity that is induced by transcription [13], [50], [51], [67], [68]. Much is known about the mechanism by which superhelical denaturation of the FUSE element regulates transcription through binding of single strand-specific regulatory proteins. Less is known about the roles of the other two alternate structures, and nothing is known about the competition among them or how it modulates transcription [67].

**Figure 6:**
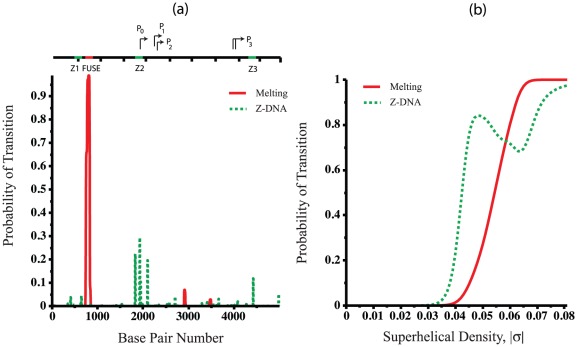
Transition behavior for human c-*myc* gene. (a) The transition profile calculated with BDZ*trans* for the human c-*myc* gene at 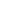 = −0.06 and T = 310 K. The upper panel located the promoters, the three *Alu*I fragments, and the FUSE element. (b) Probability of denaturation and the B-Z transition as a function of superhelical density 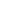 at T = 310 K.

Here we use BDZ*trans* to investigate how the strand separation and B-Z transitions compete in this region. Specifically, we analyze the transition behavior of a 5 kb region around the *c-myc* gene that includes the FUSE element and the three Z-susceptible sites. Fig. 6(a) shows the transition profile of this region calculated using BDZ*trans* at T = 310 K and 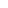 = −0.06. The upper panel in the figure marks the locations of the FUSE element and the three *Alu*I Z-sites, labeled Z1, Z2, and Z3. The locations of the promoters also are shown. Under these conditions one sees a clear melting peak at the FUSE element. For Z-DNA there are two small peaks at Z2 and Z3, and only an insignificant transition probability at Z1.

Fig. 6(b) shows the overall probabilities 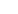 and 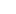 of each type of transition as a function of the superhelical density at T = 310 K. At low levels of negative superhelicity only the B-Z transition is present. As 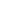 becomes more negative the probability of melting also increases, out-competing Z-DNA in some ranges. At 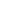
−0.06, both transitions have high probabilities of occurrence. However, as shown in Fig. 6(a) denaturation is substantially confined to the FUSE element, while the propensity to form Z-DNA is distributed among several regions, each of which has only a low transition probability. One sees that Z-formation is predicted to occur at lower superhelical densities than does FUSE melting. This suggests that the presence of the Z-forming regions will delay the onset of FUSE melting to more extreme superhelicities than would be required in their absence. Moreover, when FUSE melting occurs, it is facilitated by the partial reversion of the Z-DNA back to B-form. In this way the B-Z transitions are predicted to have regulatory effects through their modulation of FUSE melting.

### Transitions around Transcriptional Regulatory Regions

Transcription in eukaryotes has been shown to produce enough negative superhelicity to drive structural transitions in regions upstream from active genes [4]. This suggests that superhelically driven transitions to alternate DNA structures could occur there *in vivo*, where they might serve transcriptional regulatory functions. SIBZ analysis has found an enrichment of regions with Z-forming potential around transcription start sites (TSSs) [31]. Other less rigorous methods, such as *Z-Catcher* and *Z-Hunt*, have found qualitatively similar patterns of Z-DNA enrichment around TSSs [31], [55], [69], [70], although they find different numbers of sites than we do. Here we examine how superhelical B-Z transitions compete with denaturation at these locations, as well as in the regions where transcription terminates. We compare the transition properties of the TSS regions in eukaryotes with those in a prokaryote, and with those from a class of pseudogenes that are not transcribed.

#### Transcription start sites in mouse genes

**Figure 7:**
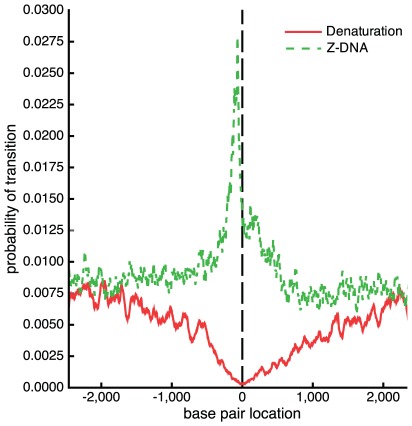
The transition probabilities for denaturation and Z-DNA at each position relative to the TSS (indicated by the vertical dashed line), averaged over 12,841 mouse genes. These profiles were calculated at 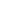 = 305 K and 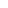 = −0.07.

First, we use BDZ*trans* to calculate the transition probability profiles of 12,841 mouse gene sequences. These are taken from a database of well characterized orthologous mouse and human gene pairs [71]. Each sequence is 5000 bp long, centered at their TSSs and oriented so they transcribe to the right. In these calculations we set 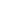 = −0.07 because this is the level of superhelicity that was experimentally found to be produced by transcription in upstream (i.e. 5′) gene flanks [4]. We also set 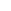 = 305 K since we find that under these conditions denaturation and B-Z transitions are approximately equally competitive remote from the TSS. Using BDZ*trans* we calculate the probability profile of each sequence for each type of transition, then average the values found at each position in the 5000 bp range over all 12,841 sequences. The results for both Z-form and denaturation are shown in Fig. 7.

One sees a clear enrichment of Z-susceptible sites in the 1 kb region surrounding the TSS, with a sharp peak just upstream of that location. The number of Z-susceptible sites levels off at distances beyond 500 bp away from the TSS, with slightly more sites predicted in the upstream, putatively intergenic region than in the downstream transcribed region.

Interestingly, the pattern for superhelically denatured sites is quite different. These sites become much less frequent in a broad region around the TSS, decreasing almost to zero in the surrounding 250 bp. The frequency of this transition increases with distance from the TSS, attaining approximately constant values around 1500 bp away. The frequency of this transition also is slightly higher in the remote regions 5′ to the TSS than it is in the distant 3′ regions. We note that these position-specific average transition probabilities tend to be low. There is no single location relative to the TSS where transition occurs in more than 2.8% of these genes. However, essentially all of these sequences experience significant levels of one or both of these transitions.

Another informative view of the transition properties of these regions is given in Table 2. There we show the numbers of genes that are found by BDZ*trans* analysis to have one or more either melted or Z-DNA regions within specified distances of the TSS. We consider four such regions: within 250 and 1000 bp upstream (i.e. 5′) of the TSS, and within 250 and 1000 bp downstream (i.e. 3′) of it. Here we assume a base pair to be in an alternate secondary structure when its probability of transition is at least 0.8, since at this point an experiment would be likely to conclude that transition has occurred. This table also compares the results computed using BDZ*trans* with those from the SIDD and SIBZ algorithms, which consider only a single transition type.

**Table 2:** This table shows the total number of mouse gene sequences containing at least one denatured (D) or Z-DNA (Z) region near their TSS at T = 305 K and 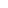 = −0.07.

Algorithm	1000 bp 5′		250 bp 5′		1000 bp 3′		250 bp 3′	
BDZ*trans*	D	Z	D	Z	D	Z	D	Z
	462	2789	34	1180	256	1954	21	618
SIDD	708		51		364		42	
SIBZ	3443		1527		2457		844	

These are sites in a given sequence where the probability of transition is at least 0.8. The regions in which these alternate structures regions are counted extend either 250 or 1000 bp downstream (3′) or upstream (5′) of the TSS. The results from the BDZ*trans*, SIDD, and SIBZ algorithms are compared.

Although the presence of transformed regions in the 5′ gene flanks is not universal, they do occur in a large number of genes. More than 25% of the genes in this data set have one or more regions of predicted alternate structure within 1000 bp upstream of their TSSs. Z-forming regions occur there in 21% of these sequences, and 42% of these are within 250 bp upstream of the TSS. In contrast, only 4% of the genes have a denaturing site within 1 kb of their TSS, and less than 10% of these are closer than 250 bp. This data also shows that more genes contain sites that are predicted to form alternate structures in the 5′ flanks of their TSSs than in their 3′ flanks. Although this is true of both Z-susceptible sites and melting regions, it is particularly clear for the B-Z transition. Nearly twice as many genes have Z-forming regions within 250 bases upstream of their TSS as have them within this distance downstream.

The SIDD and SIBZ algorithms, applied separately, do find the sites within these sequences that are susceptible to each type of transition. However, since each methods treats only one type of transition, excluding their competition, they overestimate the correct number of transforming sites, and the extent of transition at each site. This is shown in Table 2 where the results calculated by the three algorithms are compared. SIDD and SIBZ find more denatured and Z-form regions, respectively, than does BDZ*trans*, which allows these two types of transitions to compete. The total number of genes containing transformed regions found by SIDD and SIBZ together is nearly 30% larger than the number of genes containing secondary structures found using BDZ*trans*. The total number of transition is grossly overestimated by the individual algorithms, since without the presence of the competition the level of supercoiling allocated for each transition does not reflect the physical superhelicity present in the system. In other words, without competition often there are cases where a transition is predicted to occur, where in reality there is not enough supercoiling present to drive it since it was outcompeted by another transition which absorbed some of the imposed superhelicity. Since the competition among all susceptible sites is innate to the nature of stress induced transitions, it is most informative and accurate to analyze this behavior using multi-state analyses, in this case the BDZ*trans* algorithm.

As discussed earlier, denaturation in genomic DNA tends to occur in small numbers of long runs, while Z-DNA occurs in multiple shorter runs. This pattern is also seen in the present mouse gene set. In this analysis we find the average length of denatured regions to be 51.5 bp, while the average length of Z-forming regions is 25.8 bp, nearly half that of the denatured sites.

We use this set of 12,841 sequences to determine the level of competition that occurs in practice between B-Z transitions and strand separation under these conditions. First we determine the distribution of conformations that occurs at equilibrium for each sequence. Then we calculate the fraction of those conformations in which both denatured and Z-form regions are present, denoted by 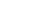. We also determine 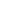 and 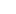, the fraction of the equilibrium population with denatured base pairs, and the fraction that contain Z-form DNA. We find that 6316 of these sequences experience substantial interaction under these conditions. (Here a sequence is regarded as experiencing a substantial level of interaction if it has 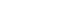, so that more than 10% of the conformations in its equilibrium distribution have both denatured and Z-form regions coexisting, and hence competing.) In 3,864 of these sequences 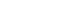, so more than half of their equilibrium conformations contain both denatured and Z-form sites. In other words, in 30.1% of the 12,841 sequences analyzed here the the presence of both Z-form and denaturation dominates the transition behavior. Of the 6,525 sequences where 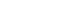 we find that denaturation dominates Z-form (i.e. 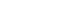) in 111 sequences, Z-form dominates denaturation (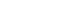) in 6396 sequences, and in 18 sequences neither transition dominates the other. (As the superhelix density and/or the temperature change, all these numbers will vary significantly.) This analysis shows that a high level of competition between these transition types occurs in many genomic DNA sequences.

#### Transcription start and end sites in human genes

Next, we contrast the transition properties near transcription start sites with those that occur around the ends of genes. In this analysis we calculate the average transition behavior using two databases composed of human genes. In the first set we analyze sequences from 14,102 human genes, obtained from the database of mouse/human orthologs [71]. As before, each sequence is 5 kb long, centered at the TSS and oriented to transcribe to the right. We also analyze a database of 27,043 sequences centered at positions where their transcripts end (TES). We identified the genomic locations of transcript ends from a database of polyadenylation signal positions in the human genome [72]. The addition of poly(A) tails to eukaryotic RNA molecules at the end of their transcription is required for subsequent nuclear export and translation, and hence is a necessary attribute of active protein coding genes.

**Figure 8:**
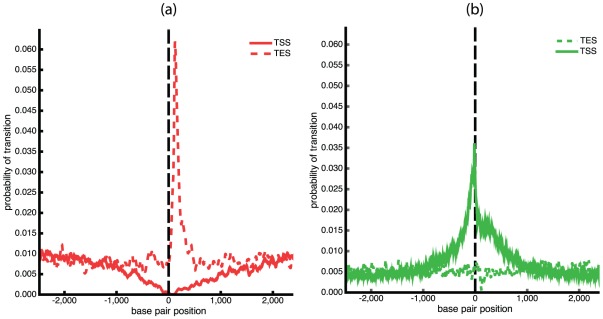
Transition profiles around gene start and end sites. The average probability for (a) denaturation and (b) Z-DNA formation are shown as functions of base pair position for human gene sequences centered at their transcription start sites (TSSs) and at their transcript end positions (TESs). These calculations were performed at T = 305 K and 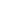 = −0.07.

We use BDZ*trans* to calculate average probabilities of denaturation and of Z-DNA formation for sites surrounding the TSSs and the TESs in these sequences. The results calculated at T = 305 K and 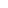 = −0.07 are shown in Fig. 8. The transition probabilities around human TSSs display the same properties as were observed above for the mouse genome. There is a sharp enhancement of Z-form regions upstream of TSSs and a broad depletion of denatured segments around these sites. This result suggests that this pattern of stress-driven transition behavior may be common to mammals, and perhaps to other eukaryotes. Previous calculations show that this qualitative pattern of Z-susceptible sites is present in higher eukaryotes [69].

The transition behavior predicted around TESs is approximately opposite to that found around TSSs. Fig. 8 shows a substantial enhancement of denatured regions immediately downstream (3′) of TES, and a slight depletion of Z-DNA there. We find that 46% of denatured sites located within 2000 bp downstream of the TES occur in the first 500 bp, indicating a clear enrichment. For Z-DNA, 24% of the transition sites that are predicted within 2000 bp downstream of the TES occur within the first 500 bp, just a slight depletion. Upstream of the TES there is no clear pattern for either transition type. A depletion of Z-DNA in the 3′-flanks of human genes has been found previously [73].

#### Transcription Start Sites in *E. coli*

In contrast to the behavior seen here in eukaryotes, it is known that in prokaryotes SIDD sites tend to cluster around promoters [74]. There has been previous evidence of depletion of Z-form DNA around TSS of prokaryotic genomes [69], [73]. Here we investigate these properties together by using the BDZ*trans* algorithm to analyze the average transition behavior of 4456 *E. coli* gene sequences. Each is 5 kb long, centered at the gene start site and oriented to transcribe to the right. Because many genes in prokaryotes are organized into operons, they may not have regulatory regions in their 5′ flanks. Many of these genes will be in close proximity to their 5′ gene neighbor, in some cases directly abutting or even overlapping it. The dataset we analyze contains sequences from all genes in the *E. coli* genome, regardless of their operon status.

**Figure 9:**
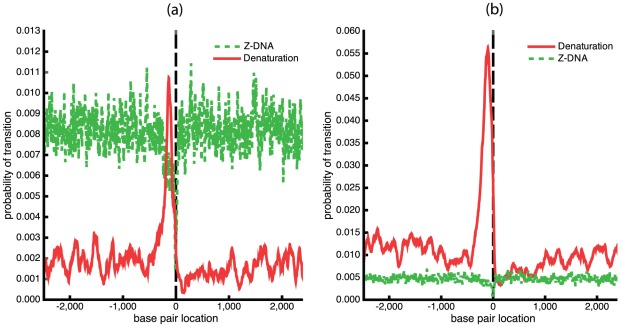
The average transition probabilities for denaturation and Z-DNA at each position relative to the gene start site were calculated at 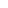 = −0.07 for 4456 *E. coli* gene sequences. The vertical dashed line indicates the gene start site, and transcription proceeds to the right. The competitive transition behavior calculated at 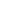 = 305 K is shown in part (a), that calculated at 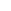 = 310 K is given in part (b).

In Fig. 9(a) we plot the average probabilities of denaturation and of Z-formation as functions of base pair location for this sequence set, calculated at T = 305 K and 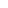 = −0.07. This analytic procedure is the same as was used above on the mouse and human sequences. In sharp contrast to what was observed in the eukaryotes, in *E. coli* we find the probability of denaturation to be enhanced 5′ to the gene start (i.e. +1) positions, and the probability of B-Z transitions to be diminished there. At this temperature a total of 753 genes contain strong Z-forming regions within 1000 bp upstream of the gene, out of which only 6.6% are within the closest 250 bp. Although only 218 denatured regions are found in the 1000 bp upstream region, 31.7% of these are within the first 250 bp. This is the opposite pattern to what is shown in Table 2 for the average transition properties in mouse. There approximately 42% and 7% of sites in the upstream 1000 bp that occur in Z-form and strand separated states, respectively, are located in the 250 bp nearest the TSS.

At this temperature the B-Z transition is predominant everywhere in the 5000 base region, except for about 250 base pairs upstream of gene start sites. Analyzing these sequences using SIDD alone overestimates the propensity for denaturation because it neglects the competing B-Z transition. The SIDD algorithm finds more than four times as many denatured regions in the 1000 bp directly 5′ of genes than does BDZ*trans*. (Data not shown.)

In Fig. 9(b) we plot the transition behavior at 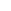 = 310 K and 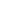 = −0.07. This is the normal human body temperature, and is known to be optimal for *E. coli* growth. This slight increase in temperature is seen to dramatically change the transition behavior of these sequences. Now denaturation dominates in the entire region, and is especially enhanced just upstream of genes. We find that about 61% of the denatured regions in the upstream 1000 bp flank are clustered in gene-proximal 250 bp of that region.

#### Mouse pseudogenes

We next analyzed a set of 4465 processed mouse pseudogenes [75]. These are pseudogenes that were created by retrotranscriptional events followed by integration, so they do not have promoters and hence are not transcribed. Although processed pseudogenes may retain a high degree of sequence similarity to their functional parent gene, they no longer experience the same selection pressures. So the analysis of this class of pseudogenes might indicate whether transition properties are retained in the absence of such pressures. Previous work has shown a decrease in the frequency of Z-susceptible sites near pseudogenes relative to active genes [73].

**Figure 10:**
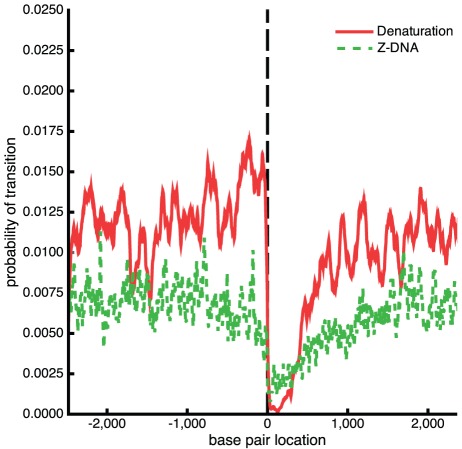
The average transition probabilities for denaturation and for Z-DNA are shown at each position relative to gene start sites for a set of 4465 mouse pseudogenes. These profiles were calculated at 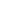 = 305 K and 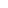 = −0.07.

As before, we analyze 5 kb sequences from these pseudogenes, centered on their first base pair and oriented so the transcriptional direction would be to the right. Their transition profiles were calculated by BDZ*trans* at 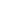 = 305 K and 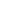 = −0.07. The transition probabilities at each position are averaged over all the sequences; the resulting profiles are shown in Fig. 10. Upstream from the start sites there is no detectable pattern for the pseudogenes, as would be expected because they lack normal 5′ flanks. Downstream, on the other hand, there is a clear depletion for both denaturing and Z-forming regions. The depletion of denaturing sites is similar to that shown by the mouse sequences in Fig. 7 and by the human sequences in Fig. 8(a), although it does not persist as far from the start position. However, the Z-susceptible sites, which are enhanced in this area in both human and mouse, are here seen to be substantially diminished. This may suggest that Z-susceptible sites in this region rapidly disappear when they are not under selection pressure. The absence of denaturing sites in this region that is seen in both normal genes and in these pseudogenes could suggest that this trait is not under strong transcriptional selection pressure. Alternatively, it could be that this property may experience differential selection pressure in these sequence types, but may respond relatively slowly to it.

## Discussion

This paper develops the first computational method to analyze the statistical mechanical equilibrium behavior of a negatively supercoiled DNA with any base sequence that is subject to multiple, competing secondary structural transitions. This method calculates the probability of each base pair transforming into any of its available alternative secondary structures, as well as the ensemble average values of other parameters of interest. The analysis of multi-state competitions is required when sites susceptible to each type of transition are present in the same topological domain, as occurs in virtually all domains of kilobase length.

The first implementation of this method is the BDZ*trans* algorithm, which analyzes the competition between superhelical strand separation and B-Z transitions. This competition was chosen for two reasons. First, complete information is available regarding the energetics of both of these transitions under comparable environmental conditions. Second, every base pair in DNA is susceptible to forming either alternate structure, at least in principle. So these two transitions compete in every DNA sequence. Using this algorithm we show that the competition between these transitions in superhelical DNA is highly intricate, depending in complex ways on base sequence, superhelix density, and temperature. Due to the temperature dependence of the energetics of strand separation, B-Z transitions dominate at low temperatures and denaturation becomes increasingly competitive as temperature increases. In the physiologically important temperature range 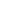
300–315 K, both types of transitions are reasonably competitive. Their interactions also depend in complex ways on the sequences and lengths of the transforming regions, and on the superhelix density. In an illustrative sample calculation we documented conditions in which B-Z transitions are preferred over denaturation at high superhelix densities, even when the temperature is above the melting temperature of A+T-rich DNA.

To determine how strand separation and B-Z transitions interact in practice in superhelical domains, we used BDZ*trans* to analyze 12,841 mouse gene sequences at 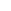 = 305 K and superhelix density 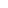 = −0.06. For each sequence in this set we assessed its equilibrium distribution, then determined the fraction of conformations in that distribution that had specific properties of interest. First, for every sequence in this set the probability of having no transition was essentially zero; virtually every conformation in the equilibrium distribution of every sequence was found to undergo some sort of transition under these conditions. Next, for each sequence we determined the frequency in its equilibrium distribution of conformations in which both denatured and Z-form sites were simultaneously present. We found that approximately half of these sequences have equilibrium distributions in which more than 10% of the molecules have coexisting Z-form and denatured regions. In 30% of the sequences these states dominate the equilibrium distribution. That is, more than half the molecules in the equilibrium distribution contain both Z-form and denatured regions. This shows the prevalence of states involving all three conformations in superhelically stressed genomic sequences, and indicates the importance of using computational methods that analyze their interactions.

We have shown that one cannot develop an accurate analysis of multistate transitions by amalgamating results from two-state techniques. To this end we compared the results from BDZ*trans* with those from SIDD and SIBZ, two-state algorithms that treat strand separation and B-Z transitions, respectively. Although the dominant transition regions are often correctly identified by the individual algorithms, they substantially overestimate both the number of such regions and their relative propensities to experience transition. This happens because each transition type in fact competes with the other, transitions to which decrease the effective level of supercoiling. A variety of examples have shown that sequences susceptible to both types of transition can exhibit particularly complex behaviors that cannot be captured by combining the results from the two-state SIDD or SIBZ analyses. In essence, this is because one cannot get an accurate depiction of an equilibrium distribution that contains many conformations in which denatured and Z-form sites coexist by mixing one distribution in which only denatured states occur with a second distribution in which only Z-forming states are present. This is why a full multi-state analysis is required to accurately depict competitions involving multiple alternate conformations in superhelical DNA.

Comparisons of the BDZ*trans* results with those from experiments investigating the superhelical competition between strand separation and B-Z transitions shows that, when the correct energetics are used, the BDZ*trans* algorithm accurately depicts the competitive transition behavior that was observed in these experiments [26].

We performed the first theoretical analysis of the competition between superhelical denaturation and B-Z transitions in the control regions of the *c-myc* oncogene, where both transitions are known to occur *in vivo*, and have been posited to serve regulatory functions [67]. Our results suggest that B-Z transitions near the *c-myc* promoters could modulate the known regulatory effects of strand separation at the upstream FUSE element. When the energetics of formation of the quadriplex that also can occur in this region become available, we will model the full three-way competitive interactions that can occur among these transitions in a quantitatively precise manner. We anticipate that this approach will illuminate the competitions among these three transitions, and thereby assist scientists to design experiments that assess their regulatory interactions.

We used BDZ*trans* to analyze the competitive transition behaviors of collections of mouse and human gene sequences. Each sequence was 5000 bp long, aligned and centered on their annotated transcription start site (TSS). We found a sharp increase of Z-forming sites that peaks just before the TSS, then continues a short distance into the transcribed region. This apparent enrichment suggests that B-Z transitions might be involved in the transcriptional regulation of some genes. Interestingly, the BDZ*trans* analysis of these mammalian gene sets also found that sites susceptible to superhelical denaturation are highly depleted over a broad region extending approximately one kilobase on either side of the TSS. The similarities of the patterns found for both transitions in human and mouse sequences suggests that these may be universal properties of mammalian genomes, and may also occur in other eukaryotes. This question will be investigated in future work.

The depletion of stress-denaturable sites around mammalian TSSs may seem surprising, as strand separation is an essential step in the initiation of transcription from every gene. However, this process is stringently regulated by interactions between the DNA and a large number of other molecules. It is possible that the occurrence of superhelically denatured sites in 5′ gene flanks could disrupt this regulation in some manner. It has been shown that transcription can be initiated by the presence of single stranded regions of DNA alone, without requiring any other regulatory factors [76]. So if a site susceptible to superhelical denaturation occurred within the first kilobase 5′ of a gene, where its transcription would produce enough negative superhelicity to drive denaturation, the resulting open region could initiate unintended additional rounds of transcription. If this were a deleterious event, sites susceptible to superhelical strand opening would be expected to be disfavored near TSSs. We note, however, that superhelical destabilizations at more remote positions are known to serve specific regulatory functions. The FUSE element that is located 2 kb upstream from the major *c-myc* promoters regulates transcription of this gene in humans by processes involving superhelical destabilization [36].

The situation may be expected to be rather different in prokaryotes. These organisms are highly gene dense, so the intergenic transcriptional regulatory machinery must be positioned close to the genes or operons they control. Also, superhelicity is not transient in prokaryotes, but is maintained within domains by enzymatic as well as by transcriptional processes. The superhelix density in *E. coli* changes with environmental conditions and growth state, and is coupled directly to the expression levels of genes that are differentially expressed under these conditions. Our results suggest that superhelical denaturation would be highly competitive with B-Z transitions at temperatures characteristic of growth phase in a host, while B-Z transitions would dominate at the lower temperatures that occur outside of a host. However, the level of negative superhelicity imposed on the genome by gyrase also is higher during growth phase than in stationary phase. So a careful analysis of this situation requires that both effects be included. This matter also will be investigated in future work.

BDZ*trans* analysis of a prokayotic gene set finds the opposite transition behavior in their 5′ flanks than was found for eukaryotes. A clear enrichment of denatured sites just upstream of the TSS (i.e. the +1 position in prokaryotic genes) has been found at 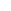 = 310 K in *E. coli*, as well as in many other prokaryotic genomes that have been analyzed previously with the SIDD algorithm. Interestingly, at the temperature where the probabilities of strand separation and of B-Z transition are comparable in eukaryotes, in *E. coli* one finds that Z-DNA dominates away from the +1 gene positions. This result suggests that fundamental differences may exist in the process of transcription as it occurs in prokaryotes and in eukaryotes.

When we compared the competitive transition behaviors around transcription start (TSS) and gene stop (TES) sites in humans, we found that opposite patterns prevail in the two regions. At the TSS the number of Z-susceptible sites is increased and the number of denaturation-susceptible sites decreased, relative to more distant regions. The opposite pattern occurs in the 3′ regions proximal to TESs. In these locations there is a clear and substantial enrichment of denaturing states, and a slight diminishing of Z-susceptible sites. This suggests that denatured DNA might play some role in processes occurring near gene 3′ flanks.

The transition properties of transcribed mouse genes have been compared to those of a set of processed pseudogenes that do not transcribe. The results obtained by BDZ*trans* show no apparent pattern for either transition upstream of the pseudogene start sites. However, there is a substantial decrease in the number of sites susceptible to either type of transition just downstream of the pseudogene “start” positions. This result suggests that the maintenance of Z-susceptible sites just 3′ of the TSS in transcribed mouse genes may be under selection pressure, disappearing when that pressure is removed.

To illustrate the practical utility of the BDZ*trans* algorithm, suppose that superhelical transition at a specific site is hypothesized to serves some regulatory function. To establish this hypothesis one must first show that the superhelical transition actually occurs at the site, and then prove that it exerts a regulatory effect. These questions are frequently investigated by inserting a segment containing the putatively regulatory susceptible site into a plasmid, perhaps along with a reporter gene. However, if superhelical transition at the test site is found not to occur in the plasmid it could be either because the hypothesis is false, or because in the plasmid that site competes with different alternatives than it does in its genomic context. Conversely, just because the transition occurs in the plasmid does not automatically mean that it also will occur in its genomic context. One can only draw inferences from the plasmid behavior regarding the genomic activity if the behaviors of the test site in the two contexts are comparable. The theoretical methods developed in this paper enable investigator to assess how the transition behavior of a site would be expected differ when it is placed in different contexts. Use of these methods will enable experimenters to design plasmids that most accurately address their questions.

After transition at the test site has been shown to occur in the plasmid, it remains to establish that it is the superhelical transition itself serves the regulatory function, and not some other attribute of the site. To do this one must alter the transition properties of the test site without changing its other attributes - in particular the local base sequence of the region involved. One can insert at a remote position on the plasmid a different susceptible site that is designed to outcompete the transition at the test site. If transition at the insert site happens first, it will delay the transition at the test site to more extreme superhelicities. If the regulatory effect is delayed to the same degree, this is strong evidence that it is indeed the superhelical transition that exerts the regulatory effect. To design experiments of this sort one needs a way to assess how various inserts would compete with a given test site within a given plasmid. The multistate analytical methods developed here will enable experimenters to make these assessments. Since B-Z transitions relax the most superhelicity per base pair, under most conditions they transform at less extreme superhelicities than do other types of transitions. So the natural choice for a competitive insert would be a Z-susceptible site. If the transition whose putative regulatory properties are being tested is either denaturation or another BZ transition, then use of the BDZ*trans* method presented in this paper will enable experimenters to design the correct systems to rigorously test their hypotheses.

These examples show how the techniques presented in this paper can be of immediate use to experimenters. Our precise quantitative method has the potential to enable the design of much more accurate and rigorous experiments than would otherwise be possible.

The multistate methods developed here are capable of treating competitions involving all the possible secondary structures that can be driven by supercoiling. In addition to the Z-form and denatured conformations, this could include G-quadriplexes, H-form DNA, cruciforms, and possibly others. However, in order to make quantitative predictions of the superhelical competitive behavior of sequences containing sites that can form these structures, their transition energetics must be known under the assumed environmental conditions. This limits the present applicability of our method to treating competitions involving B-Z transitions and denaturation, as was done here. Information is available regarding the energetics of superhelical cruciform extrusion at perfect inverted repeat sequences, and the energy costs of some types of imperfections are known [65]. So analyses that include extrusion of cruciforms are being developed. Unfortunately, sufficiently complete information regarding the energetics of forming general G-quadriplexes and H-form triplexes is not available at present. The approach presented here will become applicable to more situations as our understanding of transition energetics improves. In particular, information regarding the energetics of formation of the quadriplex at the CT site in the *c-myc* 5′ flank is expected to be available soon.

A website is available (http://benham.genomecenter.ucdavis.edu) where members of the scientific community may submit sequences of interest to them for analysis by the BDZ*trans* algorithm. The sequence must be either in FASTA format or in a file that contains sequence characters exclusively. Sequences of any length up to 10 kb may be submitted, although sequences of length around 5 kb are preferred. This site may also be used for SIDD and/or SIBZ analyses.
